# Role of Nucleotide Excision Repair in Cisplatin Resistance

**DOI:** 10.3390/ijms21239248

**Published:** 2020-12-04

**Authors:** Mingrui Duan, Jenna Ulibarri, Ke Jian Liu, Peng Mao

**Affiliations:** 1 Comprehensive Cancer Center, Department of Internal Medicine, University of New Mexico, Albuquerque, NM 87131, USA; mduan@salud.unm.edu (M.D.); eriellako@unm.edu (J.U.); 2 Department of Pharmaceutical Sciences, College of Pharmacy, University of New Mexico, Albuquerque, NM 87131, USA; KLiu@salud.unm.edu

**Keywords:** DNA damage, chemotherapy, XPA, ERCC1-XPF, CSB

## Abstract

Cisplatin is a chemotherapeutic drug used for the treatment of a number of cancers. The efficacy of cisplatin relies on its binding to DNA and the induction of cytotoxic DNA damage to kill cancer cells. Cisplatin-based therapy is best known for curing testicular cancer; however, treatment of other solid tumors with cisplatin has not been as successful. Pre-clinical and clinical studies have revealed nucleotide excision repair (NER) as a major resistance mechanism against cisplatin in tumor cells. NER is a versatile DNA repair system targeting a wide range of helix-distorting DNA damage. The NER pathway consists of multiple steps, including damage recognition, pre-incision complex assembly, dual incision, and repair synthesis. NER proteins can recognize cisplatin-induced DNA damage and remove the damage from the genome, thereby neutralizing the cytotoxicity of cisplatin and causing drug resistance. Here, we review the molecular mechanism by which NER repairs cisplatin damage, focusing on the recent development of genome-wide cisplatin damage mapping methods. We also discuss how the expression and somatic mutations of key NER genes affect the response of cancer cells to cisplatin. Finally, small molecules targeting NER factors provide important tools to manipulate NER capacity in cancer cells. The status of research on these inhibitors and their implications in cancer treatment will be discussed.

## 1. Introduction

**Figure 1:**
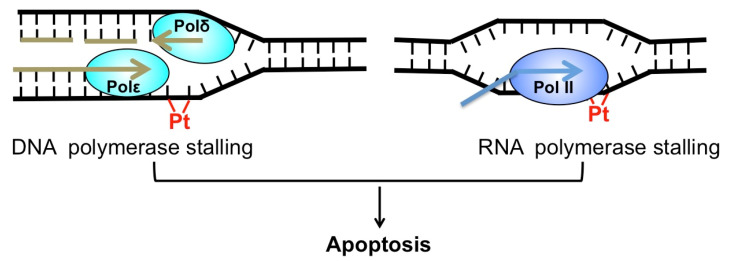
Cytotoxicity of cisplatin. Cisplatin-induced DNA adduct (red) blocks replicative DNA polymerase (**left**) and RNA polymerase (**right**). Both replication and transcription stalling can trigger apoptosis.

*Cis*-diamminedichloridoplatinum(II) (best known as cisplatin) and its derivatives, such as carboplatin and oxaliplatin, are platinum (Pt)-based chemotherapeutics [1]. Since its approval by the FDA (Food and Drug Administration) in 1978, cisplatin has been widely used to treat a number of cancers, including testicular, ovarian, bladder, lung cancer, and others [2]. Cisplatin exerts its anti-cancer activity by inducing cytotoxic DNA lesions after activation in cells by a series of aquation reactions [1]. The aquated cisplatin is highly reactive and prone to interact with a wide range of cellular substrates, including DNA. The activated cisplatin preferentially binds to two purines on the same DNA strand, causing 1,2-intrastrand crosslinks of purine bases such as Pt-d(GpG) and to a lesser extent, Pt-d(ApG) adducts [3]. These two adducts represent about 90% and 10% of cisplatin lesions, respectively [1]. Additionally, cisplatin also binds guanines on the two opposing DNA strands, inducing the formation of interstrand crosslink G-Pt-G. However, the frequency of interstrand lesion is much lower than intrastrand crosslinks. Cisplatin-induced DNA crosslinks can strongly inhibit replicative DNA polymerases and induce apoptosis [4], which explains why cisplatin selectively kills fast-proliferating cancer cells. Additionally, cisplatin adducts block elongating RNA polymerases and inhibit gene transcription [5], which also contributes to cisplatin-induced cell death (Figure 1).

The most impressive clinical success of cisplatin is the cure of over 80% patients with metastatic testicular germ cell tumors (TGCT) [6], a phenomenon that has been attributed to the intrinsic defects in DNA repair in TGCT cells [7,8]. However, the same clinical benefits are not observed for other solid tumors. Some tumor cells, including colorectal, lung, and prostate cancer cells, are intrinsically resistant to cisplatin [9]. Other tumors are originally sensitive to cisplatin, but develop resistance during the process of treatment, when tumor cells acquire further mutations to adapt to cisplatin damage. The acquired cisplatin resistance is particularly common for advanced ovarian cancer patients [10], and there is an urgent need to develop new strategies to reverse drug resistance. Resistance to platinum drugs such as cisplatin is associated with a number of cellular mechanisms, including low drug uptake through reduced expression of the copper transporter protein Ctr1 [11], inhibition of cisplatin potency by proteins such as glutathione (GSH) [12], replicative bypass of cisplatin-induced DNA damage by translesion synthesis DNA polymerases [13], and removal of cisplatin adducts by DNA repair to abolish cytotoxicity [14].

Nucleotide excision repair (NER) plays a critical role in removing helix-distorting DNA damage, including cisplatin-induced intrastrand crosslinks [15,16,17]. Previous studies in testicular tumors (i.e., TGCT) have shown low NER activity in TGCT cell extracts and low expression of several key NER proteins [7,18]. The intrinsic NER defects in TGCT cells are correlated with high cisplatin sensitivity and high cure rate among TGCT patients [19]. The impressive findings in TGCT triggered investigations in other solid tumors such as lung and ovarian cancer, in order to understand how NER contributes to cisplatin resistance in these tumors. Through extensive studies, it is now evident that elevated NER capacity in cancer cells is generally correlated with drug resistance [20,21]. The expression levels of several NER proteins (e.g., XPC, XPA, ERCC1-XPF, and XPG) and somatic mutations in XPD significantly affect cellular sensitivity to cisplatin [22,23,24]. Recent CRISPR-Cas9 screening also identified transcription-coupled NER (TC-NER) factors in resolving cisplatin-induced transcription stalling to avoid apoptosis [25]. Hence, the status of these critical NER factors represents a prospective biomarker to predict the outcome of cisplatin treatment. Furthermore, manipulation of cellular NER capacity with specific inhibitors is a potentially useful strategy to cope with cisplatin resistance.

## 2. Mechanism of NER in the Repair of Cisplatin Adducts

**Figure 2:**
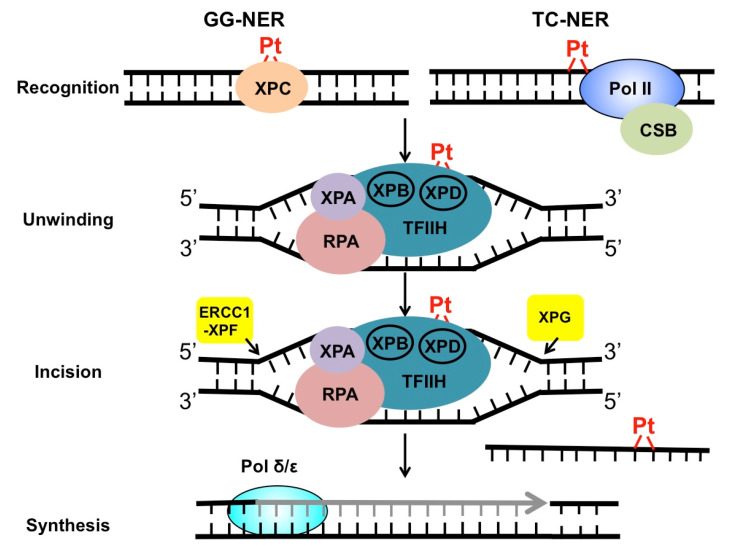
Repair of cisplatin-induced DNA damage by NER. Cisplatin damage can be recognized by surveillance protein XPC or elongating RNA Pol II, to initiate GG-NER or TC-NER. After damage recognition, the two subpathways utilize the same set of repair enzymes to conduct DNA unwinding, dual incision, and repair synthesis and ligation.

NER targets a wide range of mutagenic and cytotoxic DNA lesions. Common substrates for NER include ultraviolet (UV)-induced pyrimidine dimers, DNA adducts formed by Benzo[a]pyrene (BaP) in cigarette smoke, intrastrand crosslinks, and other helix-distorting DNA lesions [26]. The structural alteration caused by these bulky lesions interferes with DNA replication and gene transcription, and thus, needs to be repaired correctly before leading to permanent mutations or cell death. DNA helix-distorting lesions can be recognized by NER surveillance proteins such as Xeroderma pigmentosum complementation group C (XPC) and the UV-damaged DNA binding protein complex (UV-DDB) [26]. Once bound to damaged DNA, XPC recruits transcription factor II H (TFIIH), a 10-subunit protein complex consisting of two important DNA helicases, XPB and XPD. XPB and XPD, together with XPA and replication protein A (RPA), separate the two DNA strands around the damage site, creating a pre-incision DNA bubble that is recognized by repair endonucleases ERCC1-XPF and XPG. XPF and XPG cleave the damaged DNA strand on the 5′ and 3′ side relative to the damage, releasing an oligonucleotide of ~30 nucleotide (nt) containing the lesion (Figure 2). The resulting gap is filled with the activity of DNA polymerase using the undamaged strand as the synthesis template. Finally, DNA ligase I or ligase IIIα is recruited to seal the DNA backbone [26]. As damage recognition by XPC and UV-DDB can occur across the whole genome, this NER subpathway is known as global genomic NER (GG-NER). GG-NER is responsible for the removal for the majority of bulky lesions residing in the genome. NER can also be activated through the stalling of RNA polymerases. When the elongating RNA Pol II is blocked by DNA damage, the prolonged arrest of Pol II serves as a strong signal to activate transcription-coupled NER (TC-NER) [27]. TC-NER is a highly efficient repair mechanism that specifically removes transcription-impeding damage from the transcribed strand (TS) to allow transcription resumption. XPC and UV-DDB are not required for TC-NER. Instead, the stalled RNA Pol II recruits the Cockayne syndrome group B (CSB) protein, a master regulator of TC-NER [28]. CSB coordinates assembly of the downstream NER core factors for dual incision and repair synthesis. Mechanistically, GG-NER and TC-NER mainly differ in damage recognition, but share the same set of core NER enzymes for the “cut-and-patch”-type repair process (Figure 2).

Previous studies using mammalian cell extracts as well as reconstituted NER system with purified proteins have shown high activity of NER toward cisplatin-induced intrastrand crosslinks [15,29]. These in vitro cisplatin repair experiments used chemically synthesized oligonucleotides containing site-specific cisplatin adducts. Incubation of the DNA substrates with whole cell extracts or purified NER enzymes resulted in 25–29 nt excision products, due to the dual incision by XPF and XPG [15,29]. Using these well-defined NER systems, the abundance of the excision products can be detected to investigate NER activity for different types of cisplatin adducts. For example, experiments conducted in HeLa cell extracts have shown that NER enzymes exhibit slightly higher repair activity for the intrastrand crosslink Pt-d(ApG) relative to Pt-d(GpG), suggesting the local DNA sequence for the crosslink can affect repair efficiency [15]. Additionally, intrastrand adducts induced by cisplatin and its derivatives, such as oxaliplatin and JM216, can all be repaired by NER proteins with similar repair kinetics [16]. In contrast, the interstrand crosslink G-Pt-G cannot be repaired by NER [15], consistent with findings showing that interstrand crosslinks are repaired by the Fanconi anemia (FA) pathway [30]. Furthermore, published data also uncovered the regulation of cisplatin damage repair by non-NER proteins. In this regard, in vitro studies showed that the high mobility group (HMG)-domain proteins suppress excision repair of the main cisplatin adduct, Pt-d(GpG) [15,29]. HMG proteins bind to cisplatin-damaged DNA by recognizing the altered DNA structure [31], and thus, shield cisplatin adducts from NER [32]. As a result, cisplatin may cause more cytotoxicity in cancer cells with high expression of HMG proteins [33].

Repair of cisplatin damage by NER is also confirmed by experiments conducted in cells. Compared to biochemical analysis, cellular repair studies provided the advantage of analyzing both GG-NER and TC-NER in resolving cisplatin-induced DNA damage. Particularly, the development of high-throughput damage sequencing methods such as Damage-seq and eXcision Repair-seq (XR-seq) has significantly improved the understanding of cisplatin damage distribution and repair kinetics at the genome scale [17,34]. Damage-seq was built on the observation that cisplatin damage blocks replicative DNA polymerases. This method first uses a cisplatin damage-specific antibody to enrich DNA fragments containing cisplatin adducts. The damage-containing single stranded DNA is used as the template to synthesize a new strand with a replicative DNA polymerase, so that the newly synthesized strand terminates at the site of cisplatin damage. By mapping the location of the 3′ end of the nascent strand, this method allows for identification of the damage site [17]. On the other hand, XR-seq is used for high-resolution mapping of NER activity in cisplatin-treated cells. In XR-seq, the ~30-nt single-stranded excision repair fragments generated during NER are purified from cells and ligated with sequencing adaptors for next-generation sequencing [34]. By mapping the sequencing reads to the human genome and counting the number of XR-seq reads in different genomic regions, this method offers a genome-wide repair profile for cisplatin adducts as well as other bulky lesions [17]. The XR-seq data have revealed highly variable repair efficiency for cisplatin damage across the genome. Several factors, including transcription and chromatin states, can affect the repair efficiency. Particularly, the transcribed strand of active genes display robust repair by TC-NER at the early repair time points, whereas cisplatin damage located in heterochromatin is poorly repaired [17], likely due to the low access of cisplatin damage to GG-NER proteins. In contrast, Damage-seq data revealed that formation of cisplatin damage is largely uniform across the genome and mainly dictated by the underlying DNA sequences [17]. Thus, these new methods provide high-resolution maps of cisplatin damage formation and repair kinetics at the genome level, and highlight the important roles for both GG-NER and TC-NER in resolving cytotoxic damage induced by cisplatin. Indeed, as discussed below, both NER subpathways have a significant impact on the cisplatin response in cancer cells.

## 3. Roles of GG-NER Factors in Cisplatin Resistance

*XPC*: XPC is an important damage recognition factor in GG-NER [26]. XPC physically interacts with RAD23B for DNA binding and the prevention of proteasomal degradation [35]. In vitro binding data indicate that XPC-RAD23B binds with high affinity (K_D_ ∼1−3 nM) to DNA fragments containing a single cisplatin intrastrand crosslink or a single UV-induced 6,4-photoproduct [36]. The wide range of structurally diverse lesions recognized by XPC-RAD23B is accomplished by its strong binding to the thermodynamically destabilized DNA double helix caused by bulky lesions [37]. Binding of XPC to damaged DNA is critical for the recruitment of the next NER factor, TFIIH, via physical interaction with the TFIIH subunit p62 [38,39]. Hence, damage recognition by XPC represents an important rate-limiting step that largely dictates cellular GG-NER capacity. Consistent with this notion, studies performed in cancer cell lines have shown a correlation between XPC protein levels and cisplatin resistance. Overexpression of XPC in several types of cancer cells (e.g., lung, colorectal, and gastric cancers) leads to elevated cisplatin resistance [23,24,40]. On the other hand, inhibition of XPC protein production by siRNA knockdown increases cisplatin sensitivity. While the observations in cancer cell lines show a clear correlation between cisplatin sensitivity and XPC protein levels, whether or not resistance in cisplatin-treated tumors is associated with upregulated XPC has not been analyzed. Damage recognition by XPC is assisted by UV-DDB in some cases. For example, recognition of UV-induced cyclobutane pyrimidine dimer (CPD), which is less helix-distorting than 6,4-photoproduct, requires cooperation between XPC and UV-DDB [26]. UV-DDB does not appear to be required for the repair of cisplatin adducts, and overexpression of UV-DDB surprisingly sensitizes ovarian cancer cells to cisplatin by promoting apoptosis [41]. This suggests that UV-DDB may play a different role from XPC in cellular response to cisplatin damage.

*TFIIH*: Following damage recognition, the TFIIH complex is recruited to DNA to promote the subsequent repair steps. The DNA helicases XPB and XPD in TFIIH unwind damaged DNA to promote the assembly of the pre-incision complex [26] (see Figure 2). XPB is important for both transcription initiation and repair. The ATPase, but not DNA helicase activity of XPB, is required for NER [42]. In contrast, XPD appears to mainly function in NER [43]. XPD mutations that disrupt its helicase activity can significantly affect cellular NER capacity [43]. As a result, germline mutations in the *XPD* gene are associated with three severe human diseases: Xeroderma pigmentosum (XP), XP combined with Cockayne syndrome (XP/CS), and Trichothiodystrophy (TTD) [44]. Both XP and CS are known genetic disorders caused by NER defects, while TTD is linked with defective transcription [44]. Interestingly, somatic XPD mutations are frequently found in human cancers, particularly in bladder tumors [45]. A number of bladder cancer-derived XPD somatic mutations can significantly inhibit NER and strongly increase cisplatin cytotoxicity in human cells [22]. Data generated in a preclinical bladder cancer mouse model showed that XPD-deficient tumors are significantly more sensitive to cisplatin relative to XPD-proficient tumors [22]. Hence, these analyses suggest a potential role for XPD somatic mutations as a predictive marker of cisplatin treatment in bladder cancer. Furthermore, clinical studies performed in two cohorts of muscle-invasive bladder cancer patients revealed the association between XPD somatic mutations and cisplatin response [46,47]. In both studies, patients with XPD-mutated tumors had better overall survival after receiving platinum-based chemotherapy compared to XPD wild-type tumors.

*XPA*: XPA is a key scaffold protein required for both GG-NER and TC-NER. XPA itself does not possess enzymatic activities, but it plays a critical role in assembling the pre-incision complex on the damaged DNA. The N- and C-termini of XPA protein are disordered and physically interact with a variety of NER factors such as XPC, UV-DDB, TFIIH, ERCC1-XPF, and RPA [48]. The interaction between XPA and TFIIH allows the recruitment of XPA to the single-strand and double-strand DNA junction (ss/dsDNA) [49] around the damaged site created by TFIIH. Binding to the repair bubble allows XPA to properly position NER factors such as the endonuclease XPF in the right place for DNA incision [26]. As a critical regulator of NER, the XPA protein level in tumors is an important predictor for cisplatin response. XPA protein levels are significantly low in metastatic testicular tumor cells, which is consistent with positive response to cisplatin and excellent prognosis for TGCT patients [7]. In fact, supplementing cell extracts isolated from testicular tumor cells with purified XPA protein can restore the repair activity for cisplatin damage [7], implying that the low repair capacity is due to insufficient XPA protein in these cancer cells. The predictive role for XPA in cisplatin therapy is further confirmed by a survey including 207 patients diagnosed with germ cell tumors (GCTs) [50]. This study showed that GCTs patients with low *XPA* gene expression had significantly better overall survival than patients with high *XPA* expression after platinum treatment. These published data and the fact that XPA plays a central role in NER suggest that targeting XPA may be an effective strategy to improve cisplatin treatment.

*ERCC1-XPF*: ERCC1-XPF is a structure-specific endonuclease that nicks the damaged strand 5′ to the lesion during NER (Figure 2). XPF is the catalytically active subunit in the heterodimer [26]. ERCC1 does not have enzymatic activity, but it is important for recognizing ss/dsDNA junctions and interacting with XPA [51,52]. The physical interaction between ERCC1 and XPA is important for recruiting XPF to the unwound DNA to incise the damaged strand [51]. High ERCC1 expression has been linked with poor response to cisplatin therapy in a number of tumors, including non-small-cell lung carcinoma [53], head and neck [54], and ovarian cancer [55]. In contrast, low ERCC1 expression is associated with high cisplatin sensitivity, as shown in testicular tumors [18]. One molecular mechanism that suppresses *ERCC1* gene expression is DNA methylation in its promoter. Hypermethylation has been found in the *ERCC1* gene promoter in some glioma cells and is associated with suppressed *ERCC1* gene expression [56]. However, the prevalence of *ERCC1* promoter methylation in other tumor types is unknown. Furthermore, knockdown of either ERCC1 or XPF with siRNA in lung cancer cells resulted in defective repair of both intrastrand and interstrand cisplatin crosslinks, which is accompanied with increased cellular cytotoxicity [57]. Although these studies suggest that ERCC1 is an important biomarker, the reliability of ERCC1 expression alone in predicting treatment outcome has been challenged by other studies. For example, a survey in lung cancer patients showed no clear correlation between *ERCC1* mRNA levels and patient survival after cisplatin therapy [58]. While the *ERCC1* mRNA may not directly reflect its protein levels, another large-scale study using specimens obtained from 494 non-small-cell lung cancer patients failed to validate the predictive effect of immunostaining for ERCC1 protein [59], which suggests that ERCC1 protein levels alone cannot accurately predict clinical outcomes. Therefore, more detailed studies are needed to determine if the predictive role for ERCC1 only applies to a subset of tumors, or if ERCC1 should be considered together with other NER factors. One technical challenge for the analysis of ERCC1 protein in tumors is the presence of four ERCC1 isoforms. Commercial ERCC1 antibodies recognize all different isoforms [59]; however, only one isoform, ERCC1-202, is active in DNA repair [60]. Hence, a more specific antibody targeting this isoform is required to revalidate the correlation between tumor ERCC1 protein levels and cisplatin effectiveness.

*XPG*: XPG is the endonuclease that incises the damaged strand 3′ to the lesion during NER. XPG is recruited to the damaged DNA through interaction with TFIIH and it nicks DNA after 5′ incision by XPF [26]. Previous studies have shown that loss of heterozygosity of the *XPG* gene locus and reduction in *XPG* gene expression are associated with better survival for ovarian cancer patients treated with cisplatin [61]. Interestingly, the *XPG* gene promoter contains a putative CpG island and analysis of specimens obtained from ovarian cancer patients identified DNA methylation in ~20% of the tumor samples, but not in the matched blood DNA [62], suggesting that XPG expression may be influenced by epigenetic mechanisms in tumors. However, whether the methylation in *XPG* promoter is associated with better cisplatin response has not been investigated.

## 4. Roles of TC-NER Factors in Cisplatin Resistance

*CSB*: Cockayne syndrome group B (CSB) protein is the central regulator of human TC-NER [27]. CSB deficiency leads to impaired TC-NER and is associated with Cockayne syndrome, a severe neurodegenerative disorder. CSB is one of the earliest responders that bind damage-stalled RNA Pol II to initiate repair. While the detailed mechanism for CSB in initiating TC-NER remains elusive, new studies in budding yeast suggest that Rad26, a CSB homolog, utilizes the ATPase activity to evict the transcription elongation factor Spt4–Spt5 from the stalled Pol II, and thus, switches Pol II from elongation to repair [63,64]. The cellular TC-NER capacity has been shown to influence cisplatin sensitivity in cancer cells. Specifically, the reduction in TC-NER by knockdown of CSB was shown to significantly sensitize prostate and colorectal carcinoma cell lines and increase apoptosis upon cisplatin treatment, even in the absence of p53 and DNA mismatch repair [65]. The prominent cisplatin sensitivity in CSB-deficient cells is likely due to transcription blockage by cisplatin-induced DNA crosslinks [66], and the failed rescue of arrested RNA Pol II, because the prolonged blockage of Pol II has been shown as a strong signal for apoptosis [67]. CSB protein is overexpressed in a number of cancer cell lines collected from different tissues, which appears to reduce apoptosis and promote cell proliferation [68], consistent with the role for CSB in stimulating transcription elongation [69]. Considering the crucial role of CSB in TC-NER, the increased CSB expression may be linked with elevated TC-NER capacity in cancer cells. However, this hypothesis has not been tested in tumor cells. Further studies characterizing how CSB is upregulated and how its upregulation is related to cisplatin resistance will provide new insights into the connection between TC-NER and drug resistance. On the other hand, cisplatin-induced transcription stalling is harmful to non-dividing cells such as terminally differentiated healthy cells. The essential role for TC-NER in rapidly resolving transcription stress plays an important role in protecting healthy cells from the side effects of cisplatin. Indeed, it has been shown that CSB deficiency inhibits the repair of cisplatin damage in the sensory hair cells in the organ of Corti, and thus, predisposes mice to hearing loss after cisplatin treatment [70].

*CSA*: Like CSB, mutations in Cockayne syndrome group A (CSA) cause deficient TC-NER and neurological degeneration [71]. The CSA protein contains seven WD40 motifs that are required for protein–protein interaction. CSA functions as a component of the DDB1–CUL4-based E3 ubiquitin ligase complex to ubiquitylate TC-NER proteins, including CSB and the stalled RNA Pol II [72]. Additionally, CSA cooperates with CSB to recruit XPA Binding Protein 2 (XAB2), the nucleosomal binding protein HMGN1, and elongation factor TFIIS to UV-stalled RNA Pol II [73]. A published study using CRISPR-Cas9 screening identified CSA as one prominent target that protects colon adenocarcinoma cells against the clinically applied platinum drug oxaliplatin [25]. The same study also showed that CSB, ERCC1, and XPF are important for cell survival upon oxaliplatin treatment [25]. In addition to CSB and CSA, previous studies in human cells have revealed another two important TC-NER factors—UV Stimulated Scaffold Protein A (UVSSA) and Ubiquitin Specific Peptidase 7 (USP7) [71]. UVSSA interacts with RNA Pol II and recruits the deubiquitylase USP7 to the stalled Pol II to stabilize the CSB protein [74,75]. However, the roles for UVSSA and USP7 in the repair of cisplatin damage have not been characterized.

## 5. NER Inhibitors and Their Roles in Suppressing DNA Repair

Given the important role of NER in the removal of cisplatin adducts, many efforts have been made to develop small molecules targeting different NER proteins [76]. These inhibitors are useful tools for basic research as well as clinical applications to sensitize tumors to cisplatin and its derivatives.

A previous small molecule screening identified spironolactone (SP), an anti-aldosterone drug used for the treatment of heart failure and hypertension, as a specific inhibitor of XPB [77]. Studies conducted in several cell lines indicate that SP reduces cellular XPB protein by inducing its degradation [78,79]. XPB is a critical DNA helicase in TFIIH and functions in both transcription initiation and NER [26]. The rapid degradation of XPB protein in SP-treated cells has been shown to inhibit NER and increase cell sensitivity to DNA damaging agents such as UV and cisplatin [77], suggesting that SP might enhance cancer chemotherapy when combined with platinum drugs. Mechanistically, SP was shown to induce XPB phosphorylation on Ser90 by the kinase CDK7. The phosphorylated XPB is prone to poly-ubiquitylation by the SCF^FBXL18^ E3 ubiquitin ligase [78,79], followed by proteasomal degradation. SP-induced XPB degradation is reversible and XPB protein levels can be fully restored after removing the drug from the growth media.

New inhibitors for XPA have also been reported previously. A computer-aided screening using the XPA protein structure and a virtual library of small molecules identified 63 putative inhibitors targeting the DNA binding domain of XPA [80]. Biochemical analysis has revealed that one of the candidate inhibitors, X80, inhibits binding of XPA protein to single-stranded DNA and double-stranded DNA containing a cisplatin lesion [80]. Additionally, inhibitors targeting XPA–ERCC1 interaction have been identified. XPA interacts with ERCC1 to recruit ERCC1-XPF to damaged DNA. A previous study showed that the cell cycle checkpoint abrogator UCN-01 significantly weakens the interaction between XPA and ERCC1 and increases the cytotoxicity of cisplatin [81]. A virtual screening also identified putative inhibitors that disrupt XPA–ERCC1 interaction [82].

The repair endonuclease XPF can also be targeted to inhibit NER. Indeed, a high-throughput screen against ERCC1-XPF identified two compounds that exhibit robust inhibitory effect on the endonuclease activity at nanomolar concentrations [83]. The inhibition appears to be highly specific for XPF’s enzymatic activity, because they did not inhibit other endonucleases or DNA binding by ERCC1-XPF. Furthermore, the two compounds significantly potentiated sensitivity to cisplatin in cancer cells, and one of them has been shown to enhance cisplatin antitumor activity in a lung cancer xenograft model [83]. Another study using virtual screening based on the known XPF protein structure led to the identification of a compound labeled NSC 130813 that can disrupt ERCC1–XPF interaction and increase cytotoxicity to cisplatin [84]. A recent study followed this identified inhibitor and developed derivatives with enhanced inhibitory activity. One of the derivatives has been shown to be a potent inhibitor for ERCC1-XPF and suppress cellular NER activity upon UV irradiation [85].

While these small molecules can inhibit NER at different repair steps and represent important progress in developing new therapeutics to improve cisplatin efficacy, none of them have been clinically tested.

## 6. Discussion and Future Directions

The central role of NER in the repair of cisplatin-induced DNA damage makes a strong case for NER factors functioning as both predictive biomarkers and potential therapeutic targets to reverse drug resistance. As shown in Table 1, previous studies have demonstrated that many NER factors are associated with cisplatin resistance. Current evidence built on the clinical success in the treatment of testicular cancer patients with platinum-based therapy strongly indicates a correlation between defective NER and positive treatment response. Among NER factors analyzed in testis tumor cells, XPA, ERCC1, and XPF exhibited the most significant reduction compared to other types of cells [7]. These three core NER factors function downstream of damage recognition and are required for both GG-NER and TC-NER. Defective expression of the three factors likely inhibits two key steps in the NER process: the assembly of the pre-incision NER complex and repair incision on the 5′ side of the cisplatin damage (Figure 2). Although damage recognition by either XPC-RAD23B or stalled RNA Pol II may still occur, the low availability of downstream factors likely blocks processing of the damage, causing replication fork stalling and transcription stalling, both of which can lead to strong apoptosis. Hence, analyzing NER protein levels in tumors cells represents a feasible strategy to predict drug response and treatment efficacy. Whether one specific NER factor is sufficient to predict treatment outcomes or several NER factors should be considered simultaneously is currently an open question. Adding to this strategy is the identification of somatic mutations in important NER genes. Sequencing of tumor genomes has shown prevalent somatic mutations in *XPD* gene in bladder cancer patients [22]. These mutations likely disrupt XPD helicase activity and contribute to increased genome instability and tumorigenesis [86]. On the other hand, XPD somatic mutations have been shown to sensitize tumor cells to toxic cisplatin damage and represent a potential biomarker for positive cisplatin response [22]. Genome sequencing of more tumor samples offers the opportunity to identify new NER gene mutations that may help treatment decision.

While acquired cisplatin resistance is widely observed for different solid tumors, whether and how the resistance is correlated with changes in NER factors are still poorly understood. It has been shown that the overexpression of NER genes in cancer cells using expression vectors can increase cisplatin resistance (e.g., [23,24]); however, it is unknown if upregulation of these same NER genes occurs in tumors during cancer treatment. If cisplatin treatment indeed activates NER gene expression in tumors, it is important to determine what NER genes are most frequently upregulated and how their expression is elevated. Systematic analysis of protein and mRNA levels of NER factors at different stages of drug treatment may hold the key to uncover treatment-induced changes of specific NER proteins. Published studies have shown that DNA methylation plays an important role in regulating the transcription of several NER genes such as *ERCC1* and *XPG* [56,62]. Monitoring the DNA methylation status of these genes during drug treatment should also provide insights into how cisplatin changes the expression of NER genes.

## Figures and Tables

**Table 1:** NER factors with known functions in cisplatin resistance.

Name	Function	Role in Cisplatin Resistance	References
XPC	Damage recognition	Overexpression of XPC in cancer cells increases cisplatin resistance.	[23,24,40]
UV-DDB	Damage recognition	Overexpression of DDB2 sensitizes ovarian cancer cells to cisplatin.	[41]
XPB	3′-5′ helicase	Degradation of XPB by spironolactone increases cisplatin sensitivity.	[77]
XPD	5′-3′ helicase	Somatic mutations of XPD increase cisplatin sensitivity in cells and in bladder cancer patients.	[22,46,47]
XPA	Scaffolding protein	Low XPA expression correlates with increased cisplatin sensitivity in testicular tumors.	[7,50]
ERCC1	Partner of XPF	ERCC1 expression in tumor cells may predict cisplatin efficacy.	[18,53,54]
XPF	Endonuclease (5′ to lesion)	Low XPF expression correlates with high cisplatin sensitivity.	[18]
XPG	Endonuclease (3′ to lesion)	Low XPG expression is associated with good cisplatin response in ovarian patients.	[61]
CSB	TC-NER initiation	Knockdown of CSB sensitizes cancer cells to cisplatin.	[25,65]
CSA	TC-NER; E3 ligase protein	Knockout of CSA gene sensitizes human cells to oxaliplatin.	[25]

## References

[1] Dasari S., Tchounwou P.B. (2014). Cisplatin in cancer therapy: Molecular mechanisms of action. Eur. J. Pharmacol..

[2] Galluzzi L., Senovilla L., Vitale I., Michels J., Martins I., Kepp O., Castedo M., Kroemer G. (2012). Molecular mechanisms of cisplatin resistance. Oncogene.

[3] Fichtinger-Schepman A.M.J., Van der Veer J.L., Den Hartog J.H.J., Lohman P.H.M., Reedijk J. (1985). Adducts of the antitumor drug cis-diamminedichloroplatinum(II) with DNA: Formation, identification, and quantitation. Biochemistry.

[4] Siddik Z.H. (2003). Cisplatin: Mode of cytotoxic action and molecular basis of resistance. Oncogene.

[5] Todd R.C., Lippard S.J. (2009). Inhibition of transcription by platinum antitumor compounds. Metallomics.

[6] Horwich A., Shipley J., Huddart R. (2006). Testicular germ-cell cancer. Lancet.

[7] Köberle B., Masters J.R., Hartley J.A., Wood R.D. (1999). Defective repair of cisplatin-induced DNA damage caused by reduced XPA protein in testicular germ cell tumours. Curr. Biol..

[8] Einhorn L.H. (2002). Curing metastatic testicular cancer. PNAS.

[9] Galluzzi L., Vitale I., Michels J., Brenner C., Szabadkai G., Harel-Bellan A., Castedo M., Kroemer G. (2014). Systems biology of cisplatin resistance: Past, present and future. Cell Death Dis..

[10] Pokhriyal R., Hariprasad R., Kumar L., Hariprasad G. (2019). Chemotherapy Resistance in Advanced Ovarian Cancer Patients. Biomark. Cancer.

[11] Ishida S., Lee J., Thiele D.J., Herskowitz I. (2002). Uptake of the anticancer drug cisplatin mediated by the copper transporter Ctr1 in yeast and mammals. Proc. Natl. Acad. Sci. USA.

[12] Chen H.H.W., Kuo M.T. (2010). Role of Glutathione in the Regulation of Cisplatin Resistance in Cancer Chemotherapy. Met. Based Drugs.

[13] Vaisman A., Varchenko M., Umar A., Kunkel T.A., Risinger J.I., Barrett J.C., Hamilton T.C., Chaney S.G. (1998). The Role of hMLH1, hMSH3, and hMSH6 Defects in Cisplatin and Oxaliplatin Resistance: Correlation with Replicative Bypass of Platinum-DNA Adducts. Cancer Res..

[14] Rocha C.R.R., Silva M.M., Quinet A., Cabral-Neto J.B., Menck C.F.M. (2018). DNA repair pathways and cisplatin resistance: An intimate relationship. Clinics (Sao Paulo).

[15] Zamble D.B., Mu D., Reardon J.T., Sancar A., Lippard S.J. (1996). Repair of Cisplatin−DNA Adducts by the Mammalian Excision Nuclease. Biochemistry.

[16] Reardon J.T., Vaisman A., Chaney S.G., Sancar A. (1999). Efficient nucleotide excision repair of cisplatin, oxaliplatin, and Bis-aceto-ammine-dichloro-cyclohexylamine-platinum(IV) (JM216) platinum intrastrand DNA diadducts. Cancer Res..

[17] Hu J., Lieb J.D., Sancar A., Adar S. (2016). Cisplatin DNA damage and repair maps of the human genome at single-nucleotide resolution. Proc. Natl. Acad. Sci. USA.

[18] Welsh C., Day R., McGurk C., Masters J.R.W., Wood R.D., Köberle B. (2004). Reduced levels of XPA, ERCC1 and XPF DNA repair proteins in testis tumor cell lines. Int. J. Cancer.

[19] Masters J.R.W., Köberle B. (2003). Curing metastatic cancer: Lessons from testicular germ-cell tumours. Nat. Rev. Cancer.

[20] Ferry K.V., Hamilton T.C., Johnson S.W. (2000). Increased nucleotide excision repair in cisplatin-resistant ovarian cancer cells: Role of ercc1–xpf. Biochem. Pharmacol..

[21] Rosell R., Taron M., Barnadas A., Scagliotti G., Sarries C., Roig B. (2003). Nucleotide Excision Repair Pathways Involved in Cisplatin Resistance in Non-Small-Cell Lung Cancer. Cancer Control..

[22] Li Q., Damish A.W., Frazier Z., Liu D., Reznichenko E., Kamburov A., Bell A., Zhao H., Jordan E.J., Gao S.P. (2019). ERCC2 Helicase Domain Mutations Confer Nucleotide Excision Repair Deficiency and Drive Cisplatin Sensitivity in Muscle-Invasive Bladder Cancer. Clin. Cancer Res..

[23] Zhang Y., Cao J., Meng Y., Qu C., Shen F., Xu L. (2018). Overexpression of xeroderma pigmentosum group C decreases the chemotherapeutic sensitivity of colorectal carcinoma cells to cisplatin. Oncol. Lett..

[24] Pajuelo-Lozano N., Bargiela-Iparraguirre J., Dominguez G., Quiroga A.G., Perona R., Sanchez-Perez I. (2018). XPA, XPC, and XPD Modulate Sensitivity in Gastric Cisplatin Resistance Cancer Cells. Front. Pharmacol..

[25] Slyskova J., Sabatella M., Ribeiro-Silva C., Stok C., Theil A.F., Vermeulen W., Lans H. (2018). Base and nucleotide excision repair facilitate resolution of platinum drugs-induced transcription blockage. Nucleic Acids Res..

[26] Schärer O.D. (2013). Nucleotide Excision Repair in Eukaryotes. Cold Spring Harb. Perspect. Biol..

[27] Hanawalt P.C., Spivak G. (2008). Transcription-coupled DNA repair: Two decades of progress and surprises. Nat. Rev. Mol. Cell Biol..

[28] Gregersen L.H., Svejstrup J.Q. (2018). The Cellular Response to Transcription-Blocking DNA Damage. Trends Biochem. Sci..

[29] Huang J.C., Zamble D.B., Reardon J.T., Lippard S.J., Sancar A. (1994). HMG-domain proteins specifically inhibit the repair of the major DNA adduct of the anticancer drug cisplatin by human excision nuclease. Proc. Natl. Acad. Sci. USA.

[30] Deans A.J., West S.C. (2011). DNA interstrand crosslink repair and cancer. Nat. Rev. Cancer.

[31] Ohndorf U.-M., Rould M.A., He Q., Pabo C.O., Lippard S.J. (1999). Basis for recognition of cisplatin-modified DNA by high-mobility-group proteins. Nature.

[32] Awuah S.G., Riddell I.A., Lippard S.J. (2017). Repair shielding of platinum-DNA lesions in testicular germ cell tumors by high-mobility group box protein 4 imparts cisplatin hypersensitivity. PNAS.

[33] Zamble D.B., Mikata Y., Eng C.H., Sandman K.E., Lippard S.J. (2002). Testis-specific HMG-domain protein alters the responses of cells to cisplatin. J. Inorg. Biochem..

[34] Hu J., Adar S., Selby C.P., Lieb J.D., Sancar A. (2015). Genome-wide analysis of human global and transcription-coupled excision repair of UV damage at single-nucleotide resolution. Genes Dev..

[35] Ng J.M.Y., Vermeulen W., van der Horst G.T.J., Bergink S., Sugasawa K., Vrieling H., Hoeijmakers J.H.J. (2003). A novel regulation mechanism of DNA repair by damage-induced and RAD23-dependent stabilization of xeroderma pigmentosum group C protein. Genes Dev..

[36] Hey T., Lipps G., Sugasawa K., Iwai S., Hanaoka F., Krauss G. (2002). The XPC−HR23B Complex Displays High Affinity and Specificity for Damaged DNA in a True-Equilibrium Fluorescence Assay. Biochemistry.

[37] Min J.-H., Pavletich N.P. (2007). Recognition of DNA damage by the Rad4 nucleotide excision repair protein. Nature.

[38] Okuda M., Nakazawa Y., Guo C., Ogi T., Nishimura Y. (2017). Common TFIIH recruitment mechanism in global genome and transcription-coupled repair subpathways. Nucleic Acids Res..

[39] Yokoi M., Masutani C., Maekawa T., Sugasawa K., Ohkuma Y., Hanaoka F. (2000). The Xeroderma Pigmentosum Group C Protein Complex XPC-HR23B Plays an Important Role in the Recruitment of Transcription Factor IIH to Damaged DNA. J. Biol. Chem..

[40] Teng X., Fan X.-F., Li Q., Liu S., Wu D.-Y., Wang S.-Y., Shi Y., Dong M. (2019). XPC inhibition rescues cisplatin resistance via the Akt/mTOR signaling pathway in A549/DDP lung adenocarcinoma cells. Oncol. Rep..

[41] Barakat B.M., Wang Q.-E., Han C., Milum K., Yin D.-T., Zhao Q., Wani G., Arafa E.-S.A., El-Mahdy M.A., Wani A.A. (2010). Overexpression of DDB2 enhances the sensitivity of human ovarian cancer cells to cisplatin by augmenting cellular apoptosis. Int J. Cancer.

[42] Coin F., Oksenych V., Egly J.-M. (2007). Distinct roles for the XPB/p52 and XPD/p44 subcomplexes of TFIIH in damaged DNA opening during nucleotide excision repair. Mol. Cell.

[43] Kuper J., Braun C., Elias A., Michels G., Sauer F., Schmitt D.R., Poterszman A., Egly J.-M., Kisker C. (2014). In TFIIH, XPD Helicase Is Exclusively Devoted to DNA Repair. PLoS Biol..

[44] Lehmann A.R. (2001). The xeroderma pigmentosum group D (XPD) gene: One gene, two functions, three diseases. Genes Dev..

[45] Guo G., Sun X., Chen C., Wu S., Huang P., Li Z., Dean M., Huang Y., Jia W., Zhou Q. (2013). Whole-genome and whole-exome sequencing of bladder cancer identifies frequent alterations in genes involved in sister chromatid cohesion and segregation. Nat. Genet..

[46] Van Allen E.M., Mouw K.W., Kim P., Iyer G., Wagle N., Al-Ahmadie H., Zhu C., Ostrovnaya I., Kryukov G.V., O’Connor K.W. (2014). Somatic ERCC2 mutations correlate with cisplatin sensitivity in muscle-invasive urothelial carcinoma. Cancer Discov..

[47] Liu D., Plimack E.R., Hoffman-Censits J., Garraway L.A., Bellmunt J., Van Allen E., Rosenberg J.E. (2016). Clinical Validation of Chemotherapy Response Biomarker ERCC2 in Muscle-Invasive Urothelial Bladder Carcinoma. JAMA Oncol..

[48] Sugitani N., Sivley R.M., Perry K.E., Capra J.A., Chazin W.J. (2016). XPA: A key scaffold for human nucleotide excision repair. DNA Repair.

[49] Yang Z., Roginskaya M., Colis L.C., Basu A.K., Shell S.M., Liu Y., Musich P.R., Harris C.M., Harris T.M., Zou Y. (2006). Specific and Efficient Binding of Xeroderma Pigmentosum Complementation Group A to Double-Strand/Single-Strand DNA Junctions with 3′- and/or 5′-ssDNA Branches. Biochemistry.

[50] Cierna Z., Miskovska V., Roska J., Jurkovicova D., Pulzova L.B., Sestakova Z., Hurbanova L., Machalekova K., Chovanec M., Rejlekova K. (2020). Increased levels of XPA might be the basis of cisplatin resistance in germ cell tumours. BMC Cancer.

[51] Tsodikov O.V., Ivanov D., Orelli B., Staresincic L., Shoshani I., Oberman R., Schärer O.D., Wagner G., Ellenberger T. (2007). Structural basis for the recruitment of ERCC1-XPF to nucleotide excision repair complexes by XPA. EMBO J..

[52] Tsodikov O.V., Enzlin J.H., Schärer O.D., Ellenberger T. (2005). Crystal structure and DNA binding functions of ERCC1, a subunit of the DNA structure-specific endonuclease XPF–ERCC1. PNAS.

[53] Olaussen K.A., Dunant A., Fouret P., Brambilla E., André F., Haddad V., Taranchon E., Filipits M., Pirker R., Popper H.H. (2006). DNA repair by ERCC1 in non-small-cell lung cancer and cisplatin-based adjuvant chemotherapy. N. Engl. J. Med..

[54] Jun H.J., Ahn M.J., Kim H.S., Yi S.Y., Han J., Lee S.K., Ahn Y.C., Jeong H.-S., Son Y.-I., Baek J.-H. (2008). ERCC1 expression as a predictive marker of squamous cell carcinoma of the head and neck treated with cisplatin-based concurrent chemoradiation. Br. J. Cancer.

[55] Du P., Wang Y., Chen L., Gan Y., Wu Q. (2016). High ERCC1 expression is associated with platinum-resistance, but not survival in patients with epithelial ovarian cancer. Oncol. Lett..

[56] Chen H.-Y., Shao C.-J., Chen F.-R., Kwan A.-L., Chen Z.-P. (2010). Role of ERCC1 promoter hypermethylation in drug resistance to cisplatin in human gliomas. Int. J. Cancer.

[57] Arora S., Kothandapani A., Tillison K., Kalman-Maltese V., Patrick S.M. (2010). Downregulation of XPF–ERCC1 enhances cisplatin efficacy in cancer cells. DNA Repair (Amst).

[58] Booton R., Ward T., Ashcroft L., Morris J., Heighway J., Thatcher N. (2007). ERCC1 mRNA Expression Is Not Associated with Response and Survival after Platinum-Based Chemotherapy Regimens in Advanced Non-Small Cell Lung Cancer. J. Thorac. Oncol..

[59] Friboulet L., Olaussen K.A., Pignon J.-P., Shepherd F.A., Tsao M.-S., Graziano S., Kratzke R., Douillard J.-Y., Seymour L., Pirker R. (2013). ERCC1 Isoform Expression and DNA Repair in Non–Small-Cell Lung Cancer. N. Eng. J. Med..

[60] Friboulet L., Postel-Vinay S., Sourisseau T., Adam J., Stoclin A., Ponsonnailles F., Dorvault N., Commo F., Saulnier P., Salome-Desmoulez S. (2013). ERCC1 function in nuclear excision and interstrand crosslink repair pathways is mediated exclusively by the ERCC1-202 isoform. Cell Cycle.

[61] Walsh C.S., Ogawa S., Karahashi H., Scoles D.R., Pavelka J.C., Tran H., Miller C.W., Kawamata N., Ginther C., Dering J. (2008). ERCC5 is a novel biomarker of ovarian cancer prognosis. J. Clin. Oncol. Off. J. Am. Soc. Clin. Oncol..

[62] Sabatino M.A., Marabese M., Ganzinelli M., Caiola E., Geroni C., Broggini M. (2010). Down-regulation of the Nucleotide Excision Repair gene XPG as a new mechanism of drug resistance in human and murine cancer cells. Mol. Cancer.

[63] Wang D. (2020). A panorama of transcription-coupled repair in yeast chromatin. Proc. Natl. Acad. Sci. USA.

[64] Duan M., Selvam K., Wyrick J.J., Mao P. (2020). Genome-wide role of Rad26 in promoting transcription-coupled nucleotide excision repair in yeast chromatin. Proc. Natl. Acad. Sci. USA.

[65] Stubbert L.J., Smith J.M., McKay B.C. (2010). Decreased transcription-coupled nucleotide excision repair capacity is associated with increased p53- and MLH1-independent apoptosis in response to cisplatin. BMC Cancer.

[66] Damsma G.E., Alt A., Brueckner F., Carell T., Cramer P. (2007). Mechanism of transcriptional stalling at cisplatin-damaged DNA. Nat. Struct. Mol. Biol..

[67] Ljungman M., Zhang F. (1996). Blockage of RNA polymerase as a possible trigger for u.v. light-induced apoptosis. Oncogene.

[68] Caputo M., Frontini M., Velez-Cruz R., Nicolai S., Prantera G., Proietti-De-Santis L. (2013). The CSB repair factor is overexpressed in cancer cells, increases apoptotic resistance, and promotes tumor growth. DNA Repair (Amst).

[69] Selby C.P., Sancar A. (1997). Cockayne syndrome group B protein enhances elongation by RNA polymerase II. Proc. Natl. Acad. Sci. USA.

[70] Rainey R.N., Ng S., Llamas J., van der Horst G.T.J., Segil N. (2016). Mutations in Cockayne Syndrome-Associated Genes (Csa and Csb) Predispose to Cisplatin-Induced Hearing Loss in Mice. J. Neurosci..

[71] Vermeulen W., Fousteri M. (2013). Mammalian Transcription-Coupled Excision Repair. Cold Spring Harb. Perspect. Biol..

[72] Saijo M. (2013). The role of Cockayne syndrome group A (CSA) protein in transcription-coupled nucleotide excision repair. Mech. Ageing Dev..

[73] Fousteri M., Vermeulen W., Van Zeeland A.A., Mullenders L.H.F. (2006). Cockayne Syndrome A and B Proteins Differentially Regulate Recruitment of Chromatin Remodeling and Repair Factors to Stalled RNA Polymerase II In Vivo. Mol. Cell.

[74] Nakazawa Y., Sasaki K., Mitsutake N., Matsuse M., Shimada M., Nardo T., Takahashi Y., Ohyama K., Ito K., Mishima H. (2012). Mutations in UVSSA cause UV-sensitive syndrome and impair RNA polymerase IIo processing in transcription-coupled nucleotide-excision repair. Nat. Genet..

[75] Schwertman P., Lagarou A., Dekkers D.H.W., Raams A., van der Hoek A.C., Laffeber C., Hoeijmakers J.H.J., Demmers J.A.A., Fousteri M., Vermeulen W. (2012). UV-sensitive syndrome protein UVSSA recruits USP7 to regulate transcription-coupled repair. Nat. Genet..

[76] Barakat K., Tuszynski J. (2013). Nucleotide Excision Repair Inhibitors: Still a Long Way to Go. New Res. Dir. DNA Repair.

[77] Alekseev S., Ayadi M., Brino L., Egly J.-M., Larsen A.K., Coin F. (2014). A Small Molecule Screen Identifies an Inhibitor of DNA Repair Inducing the Degradation of TFIIH and the Chemosensitization of Tumor Cells to Platinum. Chem. Biol..

[78] Ueda M., Matsuura K., Kawai H., Wakasugi M., Matsunaga T. (2019). Spironolactone-induced XPB degradation depends on CDK7 kinase and SCFFBXL18 E3 ligase. Genes Cells.

[79] Kemp M.G., Krishnamurthy S., Kent M.N., Schumacher D.L., Sharma P., Excoffon K.J.D.A., Travers J.B. (2019). Spironolactone Depletes the XPB Protein and Inhibits DNA Damage Responses in UVB-Irradiated Human Skin. J. Invest. Dermatol..

[80] Neher T.M., Shuck S.C., Liu J., Zhang J.-T., Turchi J.J. (2010). Identification of novel small molecule inhibitors of the XPA protein using in silico based screening. ACS Chem. Biol..

[81] Jiang H., Yang L.-Y. (1999). Cell Cycle Checkpoint Abrogator UCN-01 Inhibits DNA Repair: Association with Attenuation of the Interaction of XPA and ERCC1 Nucleotide Excision Repair Proteins. Cancer Res..

[82] Barakat K.H., Jordheim L.P., Perez-Pineiro R., Wishart D., Dumontet C., Tuszynski J.A. (2012). Virtual Screening and Biological Evaluation of Inhibitors Targeting the XPA-ERCC1 Interaction. PLoS ONE.

[83] Arora S., Heyza J., Zhang H., Kalman-Maltese V., Tillison K., Floyd A.M., Chalfin E.M., Bepler G., Patrick S.M. (2016). Identification of small molecule inhibitors of ERCC1-XPF that inhibit DNA repair and potentiate cisplatin efficacy in cancer cells. Oncotarget.

[84] Jordheim L.P., Barakat K.H., Heinrich-Balard L., Matera E.-L., Cros-Perrial E., Bouledrak K., El Sabeh R., Perez-Pineiro R., Wishart D.S., Cohen R. (2013). Small molecule inhibitors of ERCC1-XPF protein-protein interaction synergize alkylating agents in cancer cells. Mol. Pharmacol..

[85] Elmenoufy A.H., Gentile F., Jay D., Karimi-Busheri F., Yang X., Soueidan O.M., Weilbeer C., Mani R.S., Barakat K.H., Tuszynski J.A. (2019). Targeting DNA Repair in Tumor Cells via Inhibition of ERCC1-XPF. J. Med. Chem..

[86] Kim J., Mouw K.W., Polak P., Braunstein L.Z., Kamburov A., Tiao G., Kwiatkowski D.J., Rosenberg J.E., Van Allen E.M., D’Andrea A.D. (2016). Somatic *ERCC2* mutations are associated with a distinct genomic signature in urothelial tumors. Nat. Genet..

